# The complex pattern of genetic associations of leprosy with HLA class I and class II alleles can be reduced to four amino acid positions

**DOI:** 10.1371/journal.ppat.1008818

**Published:** 2020-08-10

**Authors:** Monica Dallmann-Sauer, Vinicius M. Fava, Chaïma Gzara, Marianna Orlova, Nguyen Van Thuc, Vu Hong Thai, Alexandre Alcaïs, Laurent Abel, Aurélie Cobat, Erwin Schurr

**Affiliations:** 1 Program in Infectious Diseases and Immunity in Global Health, The Research Institute of the McGill University Health Centre, Montreal, Quebec, Canada; 2 McGill International TB Centre, McGill University, Montreal, Quebec, Canada; 3 Departments of Human Genetics and Medicine, Faculty of Medicine, McGill University, Montreal, Quebec, Canada; 4 Laboratory of Human Genetics of Infectious Diseases, Necker Branch, INSERM UMR1163, Paris, France; 5 Université de Paris, Imagine Institute, Paris, France; 6 Hospital for Dermato-Venereology, Ho Chi Minh City, Vietnam; 7 St. Giles Laboratory of Human Genetics of Infectious Diseases, Rockefeller Branch, The Rockefeller University, New York, New York, United States of America; University of Washington, UNITED STATES

## Abstract

Leprosy is a chronic disease caused by *Mycobacterium leprae*. Worldwide, more than 200,000 new patients are affected by leprosy annually, making it the second most common mycobacterial disease after tuberculosis. The MHC/HLA region has been consistently identified as carrying major leprosy susceptibility variants in different populations at times with inconsistent results. To establish the unambiguous molecular identity of classical HLA class I and class II leprosy susceptibility factors, we applied next-generation sequencing to genotype with high-resolution 11 HLA class I and class II genes in 1,155 individuals from a Vietnamese leprosy case-control sample. HLA alleles belonging to an extended haplotype from *HLA-A* to *HLA-DPB1* were associated with risk to leprosy. This susceptibility signal could be reduced to the HLA-DRB1*10:*01*~ HLA-DQA1*01:*05* alleles which were in complete linkage disequilibrium (LD). In addition, haplotypes containing *HLA-DRB3*~ HLA-DRB1*12:*02* and HLA-C*07:*06*~ HLA-B*44:03~ HLA-DRB1*07:*01* alleles were found as two independent protective factors for leprosy. Moreover, we replicated the previously associated HLA-DRB1*15:*01* as leprosy risk factor and HLA-DRB1*04:05~HLA-DQA1*03:*03* as protective alleles. When we narrowed the analysis to the single amino acid level, we found that the associations of the HLA alleles were largely captured by four independent amino acids at HLA-DRβ1 positions 57 (D) and 13 (F), HLA-B position 63 (E) and HLA-A position 19 (K). Hence, analyses at the amino acid level circumvented the ambiguity caused by strong LD of leprosy susceptibility HLA alleles and identified four distinct leprosy susceptibility factors.

## Author summary

Despite global efforts to eliminate leprosy over the past 25 years, more than 200,000 new cases are reported annually, and leprosy still represents a major public health problem in endemic regions. Leprosy presents a strong link with the host genetic background. The most significant susceptibility factors are located in the MHC region and likely involve classical HLA genes. However, the molecular identity of the HLA class I/II-leprosy risk factor(s) has been a matter of longstanding scientific dispute. By conducting a comprehensive sequenced-based analysis of HLA class I and class II genes, we are able to provide a unifying view of the complex relationship of leprosy susceptibility and HLA alleles. In addition, we show that four amino acid polymorphisms in HLA-DRβ1, HLA-B and HLA-A are sufficient to explain the majority of leprosy-HLA associations which opens the way for select protein-HLA peptide binding studies.

## Introduction

Leprosy is a chronic human disease of the skin and peripheral nerves that results from an infection with *Mycobacterium leprae*. Permanent nerve damage and disabilities can occur in leprosy patients, mainly due to delayed diagnoses and exacerbated inflammatory episodes known as type-1 reactions (T1R). Human genetic factors strongly influence susceptibility to leprosy and more than 30 loci throughout the genome have been associated with leprosy phenotypes (rev in [1]). Genome-wide association studies (GWAS) have identified single nucleotide polymorphisms (SNPs) within the Major Histocompatibility Complex (MHC) on chromosome 6p21 as the most significantly leprosy-associated genetic variants [2–7]. Divided into three classes, the MHC region harbors hundreds of genes including the classical Human Leukocyte Antigen (HLA) genes of the MHC class I and class II regions. These genes encode transmembrane receptors that present short antigenic peptides to T-cells, and, in the case of class I molecules, to NK-cells and specialized cells of the monocyte lineage (rev in [8]). The highly polymorphic class I/II genes present several bi-allelic and multi-allelic amino acid variants that impact on the strength and specificity of peptide binding. Immunologically defined HLA alleles are, on the molecular level, multi-variant haplotypes of specific genes. Molecular defined HLA alleles are now generally used for HLA genetics studies since they provide a higher resolution over immunologically–defined alleles.

Historically, a large number of HLA class I and class II alleles have been associated with leprosy or its clinical subtypes in different ethnicities (rev in [9]). Association analyses of HLA genes are challenging due to the difficulty of high-resolution allele genotyping and the complex linkage disequilibrium (LD) pattern among HLA alleles. Since earlier studies were typically conducted employing low-resolution candidate gene approaches that did not allow to adjust on the impact of complex LD pattern across the HLA region, no unifying picture of class I and class II leprosy risk alleles emerged. More recently, using HLA allele imputation, the HLA-DRB1*15:*01* allele was identified as the major driver of the MHC association signal for leprosy *per se* in the Chinese population [3, 4]. In addition to *HLA-DRB1*, variants in *HLA-DQA1* and *HLA-C* were also associated with leprosy in the Chinese population [10]. However, population-specific effects of HLA genes were suggested by two association scans conducted in Vietnamese and Indian populations that identified association signals that were independent of *HLA-DRB1* alleles and implicated a class I *HLA-C* allele [11, 12]. Hence, while the major genetic effects of leprosy susceptibility were associated with class II genes in Chinese leprosy patients it seemed possible that in Non-Chinese patients, class I alleles had a stronger effect on leprosy susceptibility.

In the present study, we used accurate HLA typing by next-generation sequencing (NGS) of three class I (*HLA-A*, *HLA-C* and *HLA-B*) and eight class II HLA genes (*HLA-DRB1*, *HLA-DRB3*, *HLA-DRB4*, *HLA-DRB5*, *HLA-DQA1*, *HLA-DQB1*, *HLA-DPA1* and *HLA-DPB1*) in a Vietnamese case-control sample. Gene-centric analyses revealed an intricate pattern of associated class I and class II alleles in line with the complex historical results. However, by taking into account allele correlations across the HLA region and by considering individual amino acid variants we showed that the complexity of associations could be reduced to the presence of specific amino acids at only four positions in the *HLA-DRB1*, *HLA-B* and *HLA-A* genes.

## Materials and methods

### Ethics statement

The study received approval from the regulatory authorities of Ho Chi Minh City, Vietnam (So3813/UB-VX and 4933/UBND-VX) and the Research Ethics Board of the Research Institute at McGill University Health Centre in Montreal, Canada (REC98-041). Written informed consent was obtained from all participants in the study. For children, assent was given by subjects, and written informed consent was obtained from parents or guardians.

### Population sample

For the present study, we enrolled 1,164 Vietnamese individuals recruited from Ho Chi Minh City in Vietnam, as previously described [7, 13, 14]. After quality control, 1,155 participants were included in the association study for leprosy *per se*, corresponding to 687 leprosy cases and 468 healthy controls (Table 1). Healthy controls were subjects living in the same districts as leprosy cases with no family history of leprosy up to third-degree relatives [7]. Due to low prevalence of leprosy, it is possible that the inclusion of genetically susceptible subjects into the group of controls will result in an underestimate of the strength of the genetic effect. The present WHO classification recognizes two leprosy subtypes: paucibacillary (PB) and multibacillary (MB) leprosy [15]. In the present dataset, 491 (71.5%) and 196 (28.5%) cases were classified as MB and PB leprosy respectively. All leprosy cases were included in the analysis of leprosy subtype using the WHO clinical classification as phenotype (Table 1). Finally, a subset of 460 borderline leprosy cases was selected for the analysis of T1R and was previously described [16]. This population sample included 230 T1R-free and 230 T1R-affected leprosy patients that were matched for gender, Ridley and Jopling subtypes and age at leprosy diagnosis (Table 1).

**Table 1:** Clinical characteristics of the Vietnamese individuals included in leprosy *per se*, leprosy subtype polarization and type-1 reaction association analyses with HLA genes.

		Leprosy *per se*		Polarization to leprosy subtypes		Type 1 reaction^b^	
		Cases	Controls	MB	PB	T1R-affected	T1R-free
**Number of samples**	**N**	687	468	491	196	230	230
**Gender**	**Male**	480 (69.9%)	257 (54.9%)	354 (72.1%)	126 (64.3%)	163 (70.9%)	159 (69.1%)
	**Female**	207 (30.1%)	211 (45.1%)	137 (27.9%)	70 (35.7%)	67 (29.1%)	71 (30.9%)
**Age**^a^ **(yr)**	Mean (SD)	19.7 (±6.7)	30.1 (±8.3)	20.2 (±6.7)	18.4 (±6.5)	19.8 (±5.5)	20.6 (±4.7)
	**Median**	20	29	21	18	20	21
**Subtype (WHO classification)**	**MB**	491 (71.5%)	-	491 (100%)	-	184 (80%)	180 (78.3%)
	**PB**	196 (28.5%)	-	-	196 (100%)	46 (20%)	50 (21.7%)
**Subtype (Ridley and Jopling classification)**	**TT**	88 (12.8%)	-	1 (0.2%)	87 (44.4%)	-	-
	**BT**	121 (17.6%)	-	14 (2.9%)	107 (54.6%)	51 (22%)	51 (22%)
	**BB**	214 (31.2%)	-	212 (43.2%)	2 (1.0%)	99 (43%)	99 (43%)
	**BL**	174 (25.3%)	-	174 (35.4%)	-	80 (35%)	80 (35%)
	**LL**	90 (13.1%)	-	90 (18.3%)	-	-	-
**Type 1 reaction**	**Yes**	244 (35.5%)	-	196 (39.9%)	48 (24.5%)	230 (100%)	-
	**No**	436 (63.5%)	-	292 (59.5%)	144 (73.5%)	-	230 (100%)
	**Unknown**	7 (1.0%)	-	3 (0.6%)	4 (2.0%)	-	-

BB: borderline-borderline; BL: borderline-lepromatous; BT: borderline-tuberculoid; LL: lepromatous-lepromatous; MB: multibacillary; PB: paucibacillary; SD: standard deviation; T1R: Type-1 reaction; TT: tuberculoid-tuberculoid; yr: years.

**a.** Age at recruitment is presented for the healthy control group in leprosy *per se* analysis, while age at leprosy diagnosis is presented in the remaining groups.

**b.** A subset of borderline leprosy cases was selected for T1R analysis, where T1R-free and T1R-affected groups were matched for age, gender and leprosy subtype.

### HLA typing by next-generation sequencing

In the present study we aimed to accurately type HLA alleles up to the second field resolution (HLA proteins). Hence, we conducted HLA typing by NGS in all 1,164 individuals for 11 HLA genes, including three class I genes–*HLA-A*, *HLA-C* and *HLA-B*–and eight loci from the class II region–*HLA-DRB1/3/4/5*, *HLA-DQA1*, *HLA-DQB1*, *HLA-DPA1* and *HLA-DPB1*. We used Holotype HLA 24/11 and 96/11 kit v2 (Omixon Biocomputing Ltd, Budapest, Hungary) to amplify the genes and generate sequencing libraries [17]. The libraries were sequenced on a MiSeq platform (Illumina, San Diego, CA, USA) in a standard flow cell using MiSeq Reagent Kit v2, 500 cycle (Illumina). The sequencing data composed of 250 bp paired-end reads was analyzed for HLA typing using Omixon HLA Twin software v2.1.4 (Omixon) with default settings and using the IPD-IMGT/HLA release 3.29 reference [18, 19]. For typing of *HLA-DRB3/4/5*, LD option was turned on for the software to use LD information in publicly available databases for determining if these loci were hemizygous. Precision of the employed method has been reported to range from 95.6% in *HLA-DQB1* to 99.53% in *HLA-A* for four-digit alleles based on data from 424 samples originated from 197 reference cell lines [20]. In 253 samples, the NGS-based HLA genotype calls have been reported to be 97.4% concordant with the high-resolution genotypes derived from a combination of Sanger-based typing and sequence‐specific primer technology [17]. In our dataset, HLA typing failed in nine out of the 1,164 sequenced samples (0.77%) and those were excluded from the study. We obtained call-rates higher than 99% for HLA class I genes, while the call-rates for class II loci ranged from 90.6% in *HLA-DQB1* to more than 98% in *HLA-DRB1/3/4/5* genes. In total, we identified 104 and 252 alleles at the first (two-digit) and second field (four-digit) resolution, respectively.

### HLA alleles and amino acid markers

For the association analyses, binary (biallelic) markers based on the presence (P) / absence (A) of each specific HLA allele or single amino acid were generated as described elsewhere [21]. These binary markers presented three possible genotypes: homozygous for the presence of the allele/amino acid (PP), heterozygous (AP) and homozygous for the absence of the allele/amino acid (AA). In the analysis of HLA alleles, binary markers were created for each two-digit and four-digit allele from the 11 HLA loci that were sequenced. In addition, we created binary markers for the presence/absence of *HLA-DRB3/4/5* genes regardless of their alleles. Gene and allele markers for *HLA-DRB3/4/5* were created using R software v3.4.4, while markers for the remaining HLA loci were generated using MakeReference command from SNP2HLA software v1.0.3 [21, 22].

HLA amino acid markers corresponded to the minor/major amino acids in biallelic protein positions (one marker per position) and binary markers for the presence/absence of each amino acid residue in multiallelic positions (three or more binary markers for single amino acid residues per multiallelic position). The amino acid markers were generated with MakeReference command from SNP2HLA software using an HLA protein reference panel with protein sequences from IPD-IMGT/HLA release 3.34 [19]. The reference panel was manually curated to include new HLA alleles detected by NGS-based typing and to complete gaps in a few known alleles from regions for which we had obtained sequencing data. The positions that we were unable to complete were coded as missing data in the MakeReference output file. A total of 491 single amino acid markers were created for the polymorphic positions in HLA-A, HLA-C, HLA-B, HLA-DRβ1 and HLA*-*DQα1 proteins.

HLA alleles or amino acids with call-rate lower than 90%, with minor allele frequency (MAF) lower than 1% in the 1,155 individuals or with deviations from Hardy-Weinberg Equilibrium in the control group with *P* < 0.01 were excluded from the analysis. In total, 198 binary markers for HLA alleles were used in the association analyses, including three markers for *HLA-DRB3/4/5* genes, 72 two-digit alleles and 123 four-digit HLA alleles. In the amino acid analysis, 424 markers passed filtering and were included in the association analyses. The tested amino acid markers corresponded to 192 biallelic amino acid changes and 232 binary markers for single residues in 71 multiallelic protein positions. The genotypes of these alleles and amino acid binary markers are available in the S1 Data.

### Statistical analyses

To account for the strong correlation caused by linkage disequilibrium (LD) among HLA alleles and among HLA amino acid markers, GEC (Genetic Type I Error Calculator) v0.2 was used to find the effective number of independent tests [23]. For that, the genomic positions of all markers were coded as being consecutives to avoid exclusion of markers that were at the same position. Although efficiency of programs for estimation of the effective number of independent tests has not been demonstrated specifically for HLA alleles/amino acids binary markers, we selected to use GEC as it has been shown to be robust to variable LD patterns across the genome [23]. Using GEC, the 198 HLA alleles markers were determined to be equivalent to 121.41 independent tests in the total population sample. Bonferroni correction for multiple testing was applied using the effective number of allele markers. This yielded a significance *P* cut-off of 4.12 × 10^-4^ (0.05/121.41), which was used in the univariable association analysis of HLA alleles. The effective number of independent amino acid tests was determined as 178 using GEC software. Hence, *P* < 2.81 × 10^−4^ (0.05/178) was used as multiple-testing cut-off in the univariable analysis of single amino acids. Univariable association analyses of HLA alleles or amino acids and leprosy susceptibility were done by logistic regression under an additive model as implemented in PLINK v1.9 [24]. For that, the three genotypes of the biallelic/binary markers (e.g. AA, AP and PP of an HLA allele marker) were coded as 0, 1 and 2 to reflect the minor allele dosage. In this model, the direction of the regression coefficient [presented as odds ratio (OR)] represented the effect of each additional minor allele. Forward conditional association analysis and pairwise reciprocal conditional analysis were also done in the additive model using PLINK, by the inclusion of the conditioned marker(s) into the model as covariate(s). In the conditional analyses, the OR_conditional_ represented the effect of each additional minor allele of the tested marker whilst controlling for the covariate(s). *P*_conditional_ < 0.01 was used as cut-off for independent association signals in the multivariable analyses. When the tested marker and a covariate presented collinearity due to complete LD, results were reported as not available (NA). Gender distribution between cases and controls was tested using Pearson's Chi-squared test with Yates' continuity correction. We included gender as a covariate in the univariable and multivariable association analyses for leprosy *per se*, since it is a known leprosy risk factor that was differently distributed between cases and controls (*P*_Chi-square_ = 3.84 × 10^−7^, Table 1). To assess the distribution of HLA alleles among leprosy cases based on two disease endophenotypes, we used logistic regression under an additive model with no covariate included, where MB were compared to PB cases and T1R-affected patients were compared to T1R-free leprosy cases [15].

We used Akaike Information Criterion (AIC), a model selection method, to compare the allele-based and amino acid-based models and select the model that best represented the data [25]. AIC was calculated for a subset of 1,136 samples with no missing genotypes among six tag allele and amino acid markers (98% of the samples), using the LOGISTIC procedure in SAS v 9.4 (SAS Institute, Cary, North Carolina, USA). The model presenting the lowest AIC (AIC_min_) was selected as presenting the best support among the tested models. Delta (Δ) AIC was calculated by subtracting the AIC of each model by the AIC_min_ to compare the two models. Models with Δ AIC of 0–2, 3–9 and ≥10 were considered to present substantial support, considerably less support and no additional support regarding the best model, respectively [26].

### Haplotype structure and LD

For LD analysis of associated markers, pairwise correlation coefficient *r*^*2*^ and D’ were calculated in healthy controls using PLINK v1.9. Phased haplotype between HLA amino acids from the same gene was determined by the NGS-based HLA typing method. For markers in different loci, we used Beagle v 3.0.4 to estimate the phased haplotypes [27]. OR and *P*-values for the haplotypic analyses were calculated using epitools R package v 0.5–10.1 [28]. Finally, Disentangler software was used for the haplotype structure visualization of the HLA genes presenting at least one four-digit allele associated with leprosy *per se* [29, 30].

### 3D visualization of HLA molecules

To identify the location of the four key leprosy-associated HLA amino acids in the protein structure, the three-dimensional structure of the HLA molecules was analyzed using UCSF Chimera v 1.13.1. [31]. HLA-DR, HLA-B and HLA-A structures were based on Protein Data Bank (PDB): 3PDO, 1SYS and 3UTQ, respectively [32–34]. These entries correspond to *HLA-DR1*, HLA-B*44:*03* and HLA-A*02:*01* alleles respectively.

## Results

### HLA allele association with leprosy

We conducted HLA genotyping by NGS of 11 HLA genes in 687 leprosy cases and 468 healthy controls from Vietnam (Table 1). In the first part of our study, 198 HLA alleles were tested for association with leprosy *per se* (S1 Table). We found 20 HLA alleles in the *HLA-A*, *HLA-C*, *HLA-B*, *HLA-DRB3*, *HLA-DRB1*, *HLA-DQA1*, *HLA-DQB1* and *HLA-DPB1* genes to be significantly associated with leprosy *per se* under an additive model (Table 2). As previous studies had reported HLA alleles to be associated with leprosy clinical subtypes (rev in [9]), we analyzed the allele distribution between MB and PB leprosy patients and between T1R free and affected cases (Table 1). We observed no significant differences of HLA alleles for MB-PB leprosy clinical subtype or T1R state (S1 Table).

**Table 2:** HLA alleles significantly associated with risk or protection from leprosy *per se* in Vietnam.

MHC region	Resolution	HLA allele	HLA allele frequency		Univariable analysis ^a^			Signal #
			Leprosy cases	Healthy controls	OR	(95% CI)	*P*-value ^b^	
Class II	Four-digit	HLA-DQA1*01:*05*	12.7%	4.5%	3.11	(2.16–4.45)	7.61 × 10^−10^	1
Class II	Four-digit	HLA-DRB1*10:*01*	12.6%	4.5%	3.08	(2.15–4.42)	1.02 × 10^−9^	1
Class II	Two-digit	HLA-DRB1*10	12.6%	4.5%	3.08	(2.15–4.42)	1.02 × 10^−9^	1
Class I	Four-digit	HLA-C*15:*05*	10.4%	3.6%	3.08	(2.08–4.58)	2.30 × 10^−8^	1
Class I	Four-digit	HLA-B*07:*05*	10.3%	3.6%	3.09	(2.08–4.60)	2.41 × 10^−8^	1
Class II	Four-digit	HLA-DQB1*05:*01*	18.7%	9.3%	2.25	(1.68–3.02)	5.26 × 10^−8^	1
Class I	Two-digit	HLA-C*15	14.0%	6.5%	2.29	(1.68–3.12)	1.34 × 10^−7^	1
Class I	Two-digit	HLA-B*07	13.5%	6.6%	2.20	(1.61–2.99)	6.52 × 10^−7^	1
Class II	Two-digit	HLA-DQA1*01	43.1%	32.8%	1.56	(1.31–1.87)	8.34 × 10^−7^	1
Class I	Four-digit	HLA-A*29:*01*	10.9%	5.6%	2.10	(1.49–2.95)	2.09 × 10^−5^	1
Class I	Two-digit	HLA-A*29	10.9%	5.6%	2.10	(1.49–2.95)	2.09 × 10^−5^	1
Class II	Two-digit	HLA-DQB1*05	29.9%	21.6%	1.55	(1.25–1.92)	7.11 × 10^−5^	1
Class II	Two-digit	HLA-DPB1*104	6.6%	2.5%	2.67	(1.65–4.34)	7.14 × 10^−5^	1
Class II	Four-digit	HLA-DPB1*104:*01*	6.5%	2.5%	2.64	(1.63–4.29)	8.81 × 10^−5^	1
Class I	Four-digit	HLA-C*07:*06*	1.2%	3.9%	0.32	(0.18–0.57)	9.95 × 10^−5^	2
Class I	Four-digit	HLA-B*44:*03*	1.3%	3.7%	0.35	(0.20–0.61)	2.51 × 10^−4^	2
Class I	Two-digit	HLA-B*44	1.5%	3.8%	0.37	(0.22–0.64)	3.83 × 10^−4^	2
Class II	Two-digit	HLA-DRB1*12	23.1%	29.7%	0.69	(0.57–0.84)	1.80 × 10^−4^	3
Class II	Four-digit	HLA-DRB1*12:*02*	23.0%	29.4%	0.69	(0.57–0.84)	2.59 × 10^−4^	3
Class II	Gene	*HLA-DRB3*	39.9%	47.2%	0.72	(0.60–0.86)	3.78 × 10^−4^	3

CI: confidence interval; OR: odds ratio.

**a)** Results in the additive model adjusted for gender. OR (95% CI) refer to the presence of the allele.

**b)** *P*-value threshold for multiple testing was set as 4.12 × 10^−4^ for the univariable analysis of HLA alleles.

**Fig 1:**
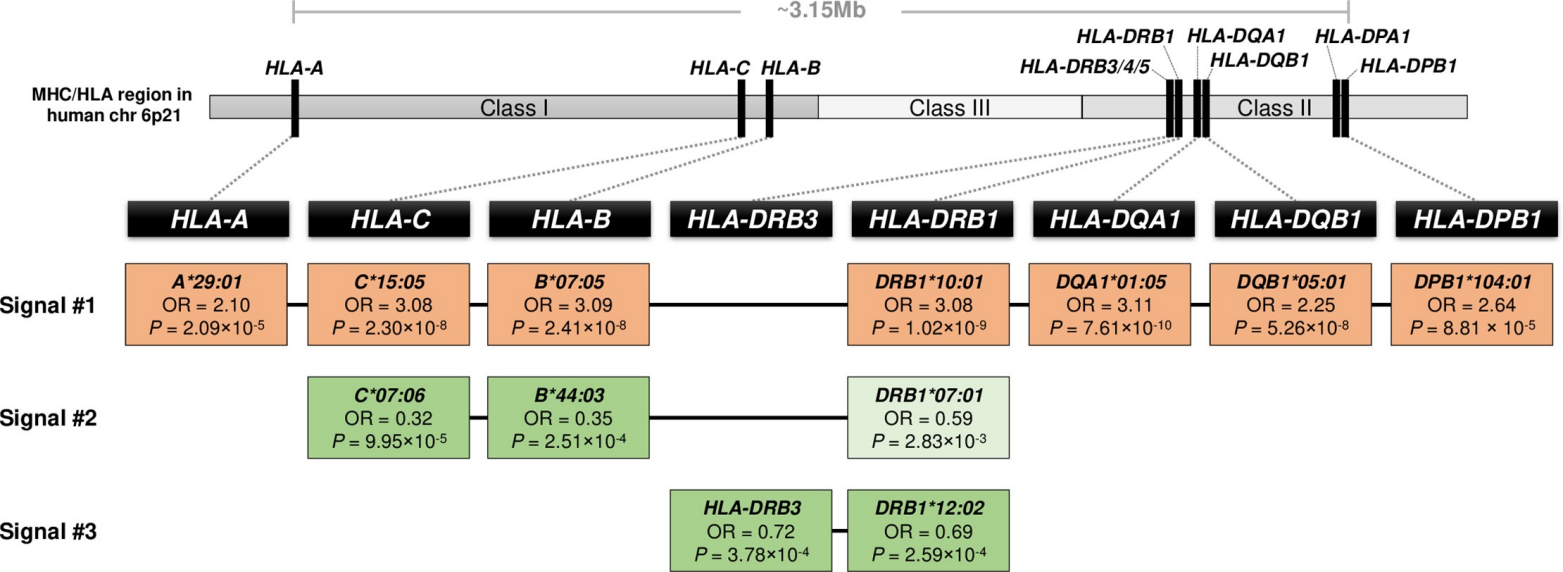
HLA class I and class II alleles with study-wide significance for association with leprosy. A schematic representation of the MHC/HLA region highlighting the genes sequenced in the present study is shown on top. The four-digit HLA alleles associated with risk and protection from leprosy *per se* are shown in orange and green, respectively. The *HLA-DRB1* allele with suggestive significance captured by the alleles in signal #2 is indicated by a box with lighter color. Odds ratios (OR) and *P*-values presented in the boxes refer to the results of univariable association analyses under an additive model (Table 2 and S1 Table). HLA alleles that belong to the same association signal based on the forward and pairwise conditional analyses are connected by lines (S2 and S3 Tables). The signals indicated common haplotypes of the corresponding alleles (S1 Fig).

Among the leprosy *per se* associated HLA alleles, HLA-DQA1*01:*05* presented the most significant evidence of association (OR = 3.11, *P* = 7.61 × 10^−10^) followed by HLA-DRB1*10:*01* (OR = 3.08, *P* = 1.02 × 10^−9^, Table 2). Of note, the HLA-DRB1*10:*01* and HLA-DQA1*01:*05* alleles were statistically equivalent due to complete LD (*r*^*2*^ = 1). The small difference in their strength of association was due to differences in missing genotypes. To test for leprosy associations independent of HLA-DRB1*10:*01*~ HLA-DQA1*01:*05*, we conducted a forward conditional association analysis of the 20 significantly associated alleles until markers presented *P*_conditional_ ≥ 0.01. When HLA-DQA1*01:*05* entered as first independent genetic variable in the model, all HLA leprosy risk alleles lost significance (S2 Table). This indicated that the associations of risk alleles were almost entirely captured by HLA-DRB1*10:*01*~ HLA-DQA1*01:*05*. We analyzed the haplotype structure of the four-digit HLA alleles and found that the HLA class I and class II leprosy risk alleles belonged to a long-range haplotype spanning from *HLA-A* to *HLA-DPB1*, with a frequency of 4.37% in cases and 1.5% in controls (haplotype HLA-A*29:01~ HLA-C*15:05~ HLA-B*07:05~ HLA-DRB1*10:01~ HLA-DQA1*01:05~ HLA-DQB1*05:01~ HLA-DPB1*104:*01*, Fig 1 and S1 Fig).

After adjusting on the effect of HLA-DQA1*01:*05* in the forward conditional analysis, significant associations were still observed for the alleles associated with protection from leprosy and the lowest *P*-value was found for the protective class I allele HLA-C*07:*06* (OR_conditional_ = 0.33, *P*_conditional_ = 2.35 × 10^−4^, S2 Table). When HLA-C*07:*06* entered in the forward conditional analysis, HLA-B*44:*03* and HLA-B*44 lost significance while a residual effect was still observed for the remaining protective alleles (S2 Table). HLA-B*44:*03* and HLA-C*07:*06* were in strong LD (*r*^*2*^ = 0.97) and statistically equivalent in pairwise multivariate analysis (S3 Table). Hence, the second independent association signal was due to HLA-C*07:*06* ~ HLA-B*44:*03* (Fig 1). After adjustment for HLA-DQA1*01:*05* and HLA-C*07:*06*, the two-digit class II allele HLA-DRB1*12 presented the lowest conditional *P*-value (OR_conditional_ = 0.71, *P*_conditional_ = 1.01 × 10^−3^, S2 Table). The association signal of HLA-DRB1*12 was primarily driven by the common HLA-DRB1*12:*02* allele which had a statistically equivalent effect with the presence of the *HLA-DRB3* gene (S3 Table) making *HLA-DRB3* ~ HLA-DRB1*12:*02* the third independent association signal in our dataset (Fig 1).

### HLA amino acid associations with leprosy

**Fig 2:**
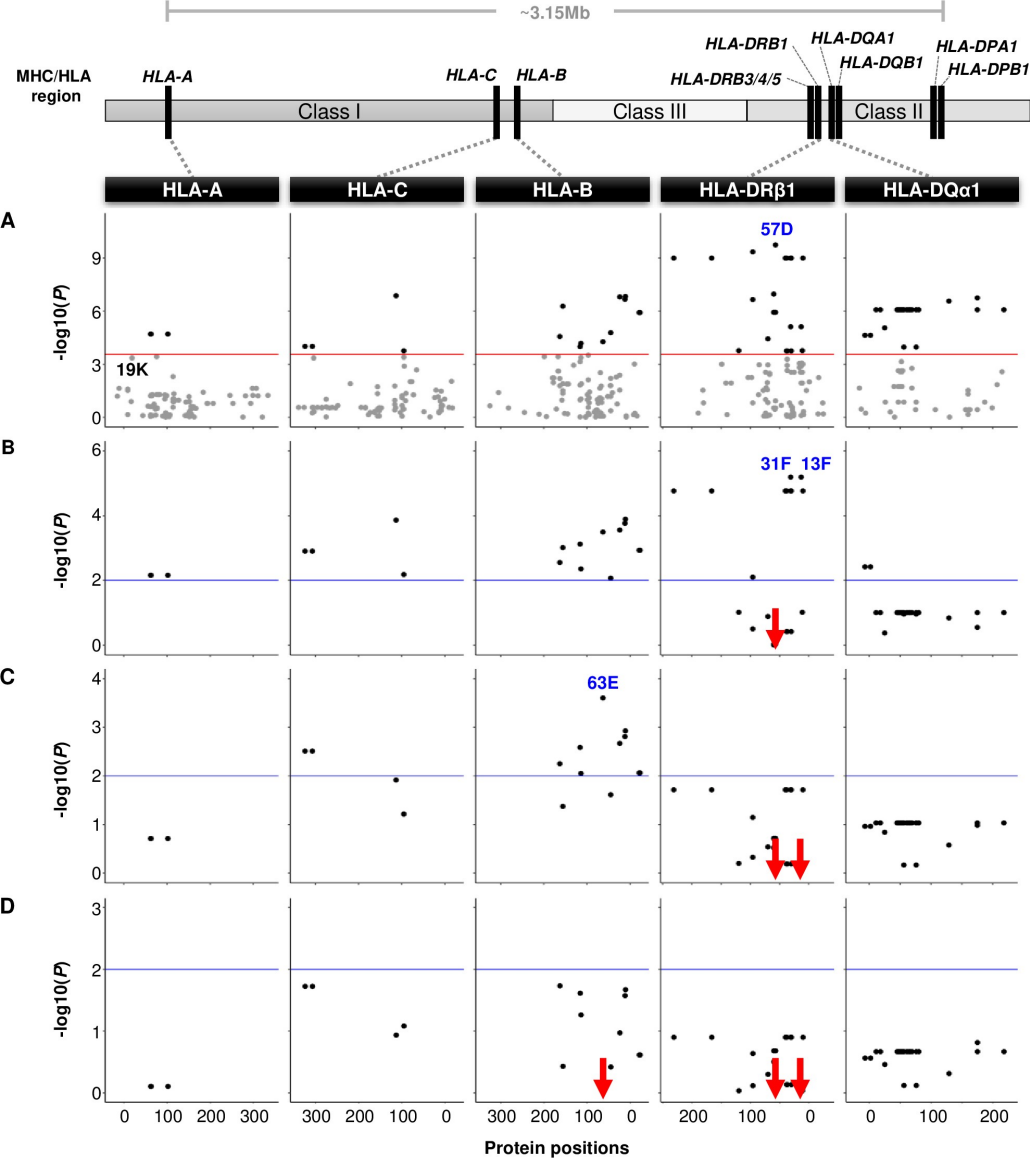
Forward conditional analysis of HLA amino acids with study-wide significance for association with leprosy. The figure presents the results for the genotypic association test under an additive model adjusted for gender and **A)** unconditioned for any marker (univariable analysis, S4 Table), **B)** conditioned on HLA-DRβ1 57D (forward step 1), **C)** conditioned on HLA-DRβ1 57D and HLA-DRβ1 13F (forward step 2) and **D)** conditioned on HLA-DRβ1 57D, HLA-DRβ1 13F and HLA-B 63E (forward step 3). A schematic representation of the MHC/HLA region highlighting the genes sequenced in the present study is shown on top. In the graphs, the y axes indicate the negative log10 of the *P*-value for association of amino acids with leprosy *per se* and the x axes correspond to the amino acid position in the mature HLA proteins. The results for each HLA gene are shown by column and the dots represent single amino acids. The HLA loci and amino acid markers are ordered based on their genomic positions. The red line corresponds to the significance *P*-value threshold for multiple-testing of amino acid markers (*P* = 2.81 × 10^−4^) in the univariable analysis (**A**) and the blue lines represent a *P*_conditional_ of 0.01 in the multivariable analyses (**B-D**). Dots shown in black reached the multiple testing *P*-value threshold in the univariable analysis while grey dots were not significant (**A**). Only amino acids significantly associated in the univariable step (**A**) were included in the multivariable analyses (**B-D**). The most significant association signal at each step is indicated by the name of the amino acid(s) shown in blue. Borderline-associated HLA-A 19K is presented in black in step (**A**). The locations of the markers used for stepwise conditioning are indicated by red arrows.

To disentangle the HLA association signals and assess the contribution of individual HLA amino acid polymorphisms, we tested if specific amino acid substitutions in HLA proteins, rather than the multi-amino acid HLA alleles, were associated with leprosy. A total of 424 single amino acids belonging to the HLA-A, HLA-C, HLA-B, HLA-DRβ1 and HLA*-*DQα1 proteins were selected for this analysis (S4 Table). In univariable analyses, 64 amino acids at 55 protein positions were significantly associated with leprosy *per se* (Fig 2A and S4 Table). The presence of aspartic acid at HLA-DRβ1 position 57 (HLA-DRβ1 57D, OR = 1.81, *P* = 1.81 × 10^−10^) was the most significant association (Fig 2A). In pairwise reciprocal conditional analysis for HLA-DRβ1 57D versus each of the remaining 63 amino acids, a significant residual effect was consistently detected for HLA-DRβ1 57D (*P*_conditional_ ≤ 5.47 × 10^-3^). This observation indicated a unique contribution of this amino acid to leprosy risk.

To test for leprosy associations independent of HLA-DRβ1 57D among the amino acids with study-wide significance, we used forward conditional regression until amino acids reached *P*_conditional_ ≥ 0.01 (Fig 2B–2D). When we adjusted on HLA-DRβ1 57D, the strongest remaining risk signal was the presence of phenylalanine at HLA-DRβ1 position 13 (HLA-DRβ1 13F, OR_conditional_ = 1.69, *P*_conditional_ = 6.49 × 10^−6^, Fig 2B). However, HLA-DRβ1 13F and phenylalanine at HLA-DRβ1 position 31 (HLA-DRβ1 31F) were in complete LD (*r*^*2*^ = 1) since presence of HLA-DRβ1 13F was always in haplotype with absence of HLA-DRβ1 31F and vice-versa. As a consequence of the inverse relationship between phenylalanines at positions 13 and 31, presence of 31F was associated with protection from leprosy (OR_conditional_ = 0.59, *P*_conditional_ = 6.49 × 10^−6^). When we adjusted for risk HLA-DRβ1 57D and HLA-DRβ1 13F, all remaining class II amino acid markers in HLA-DRβ1 and HLA-DQα1 lost evidence of association (Fig 2C). Conversely, significant residual associations were still observed for HLA-C and HLA-B amino acids. At this step, glutamic acid at HLA-B position 63 displayed the lowest *P*-value (HLA-B 63E, OR_conditional_ = 0.72, *P*_conditional_ = 2.50 × 10^−4^). Inclusion of HLA-DRβ1 57D, HLA-DRβ1 13F and HLA-B 63E in the third step of the forward conditional analysis accounted for the full association signal of the remaining 61 HLA amino acid markers at *P*_conditional_ ≥ 0.01 (Fig 2D). Interestingly, HLA-B position 63 and HLA-DRβ1 positions 13 and 57 are located in the peptide-binding grooves of the HLA-B and HLA-DRβ1 proteins (S2A and S2B Fig).

### Additional HLA alleles and amino acids involved in leprosy risk

We further investigated the presence of additional independent signals, that were missed by the stringent study-wide significance cut-offs. For this, we adjusted each of the formally non-significant HLA alleles on the presence of HLA-DQA1*01:*05*, HLA-C*07:*06* and HLA-DRB1*12 and identified seven HLA four-digit alleles with evidence (*P*_conditional_ < 0.01) for association with leprosy (S5 Table). Among the seven alleles, HLA-A*11:*02*, HLA-B*38:*02*, HLA-DRB1*15:*01* and HLA-DRB1*04:*05*~HLA-DQA1*03:*03* (both with *r*^*2*^ = 0.87) had displayed a trend toward association in the univariable analysis with *P*-values ranging from 2.56 × 10^−3^ to 4.38 × 10^−4^ (S1 and S5 Tables). In the amino acid conditional analyses, lysine in HLA-A position 19 (HLA-A 19K) was the only amino acid that remained with a *P*_conditional_ lower than 0.01 after removing the effect of HLA-DRβ1 57D, HLA-DRβ1 13F and HLA-B 63E (OR_conditional_ = 0.55, *P*_conditional_ = 8.07 × 10^−3^). HLA-A 19K presented a trend toward association as a protective amino acid in the univariable analysis (*P* = 4.49 × 10^−4^, Fig 2A).

### Comparison of HLA allele-based and amino acid-based models

Next, we tested to what extent the amino acids captured the leprosy association of the HLA alleles. Except for HLA-A*11:*02*, the association signals of the study-wide significant and borderline-significant HLA alleles were accounted for when the analysis was adjusted on the HLA-DRβ1 57D, HLA-DRβ1 13F and HLA-B 63E amino acids (S5 and S6 Tables). However, to capture the association of HLA-A*11:*02* it was necessary to include HLA-A 19K in the conditional analysis (S5 Table). HLA-A 19K is located outside the protein-binding groove (S2C Fig) and is found exclusively in the HLA-A*11:*02* allele. Taking together, these results suggested that the four amino acids offered a good explanation for the observed HLA allele associations. To further validate this conclusion, we used the AIC to select the best multivariate model from the HLA allele and amino acid analyses. The four-amino acid model with gender, HLA-DRβ1 57D, HLA-DRβ1 13F, HLA-B 63E and HLA-A 19K presented the lowest AIC among the tested models (model A in Table 3). Conversely, the models including the three significant amino acids (gender + HLA-DRβ1 57D + HLA-DRβ1 13F + HLA-B 63E, model C) and the three significant HLA alleles (gender + HLA-DQA1*01:*05* + HLA-C*07:*06* + HLA-DRB1*12, model D) presented considerably less support (Δ AIC of 5 and 12, respectively; Table 3). Indeed, even after inclusion of HLA-A*11:*02* in the four-allele model (model B), the four-amino acid model still provided the best explanation of the data (Δ AIC = 4, Table 3). Hence, the four-amino acid model was the model best fitting the data.

**Table 3:** Comparison of HLA allele-based and HLA amino acid-based models using the Akaike Information Criterion (AIC).

Models		df	Chi-Square (SCORE)	AIC	Δ AIC ^a^
**A**	Gender + HLA-DRβ1 57D + HLA-DRβ1 13F + HLA-B 63E + HLA-A 19K	5	105.2	**1436**	0
**B**	Gender + HLA-DQA1*01:*05* + HLA-C*07:*06* + HLA-DRB1*12 + HLA-A*11:*02*	5	100.6	1440	4
**C**	Gender + HLA-DRβ1 57D + HLA-DRβ1 13F + HLA-B 63E	4	98.7	1441	5
**D**	Gender + HLA-DQA1*01:*05* + HLA-C*07:*06* + HLA-DRB1*12	4	91.0	1448	12

AIC: Akaike Information Criterion, df: degrees of freedom.

**a)** Delta (Δ) AIC presents the difference between the AICs of each model with the model with the lowest AIC (in bold). Δ AIC of 0–2 represents substantial support, 3–9 shows considerably less support and models with Δ AIC ≥10 presents essentially no support.

### Haplotype analysis of HLA amino acids

**Fig 3:**
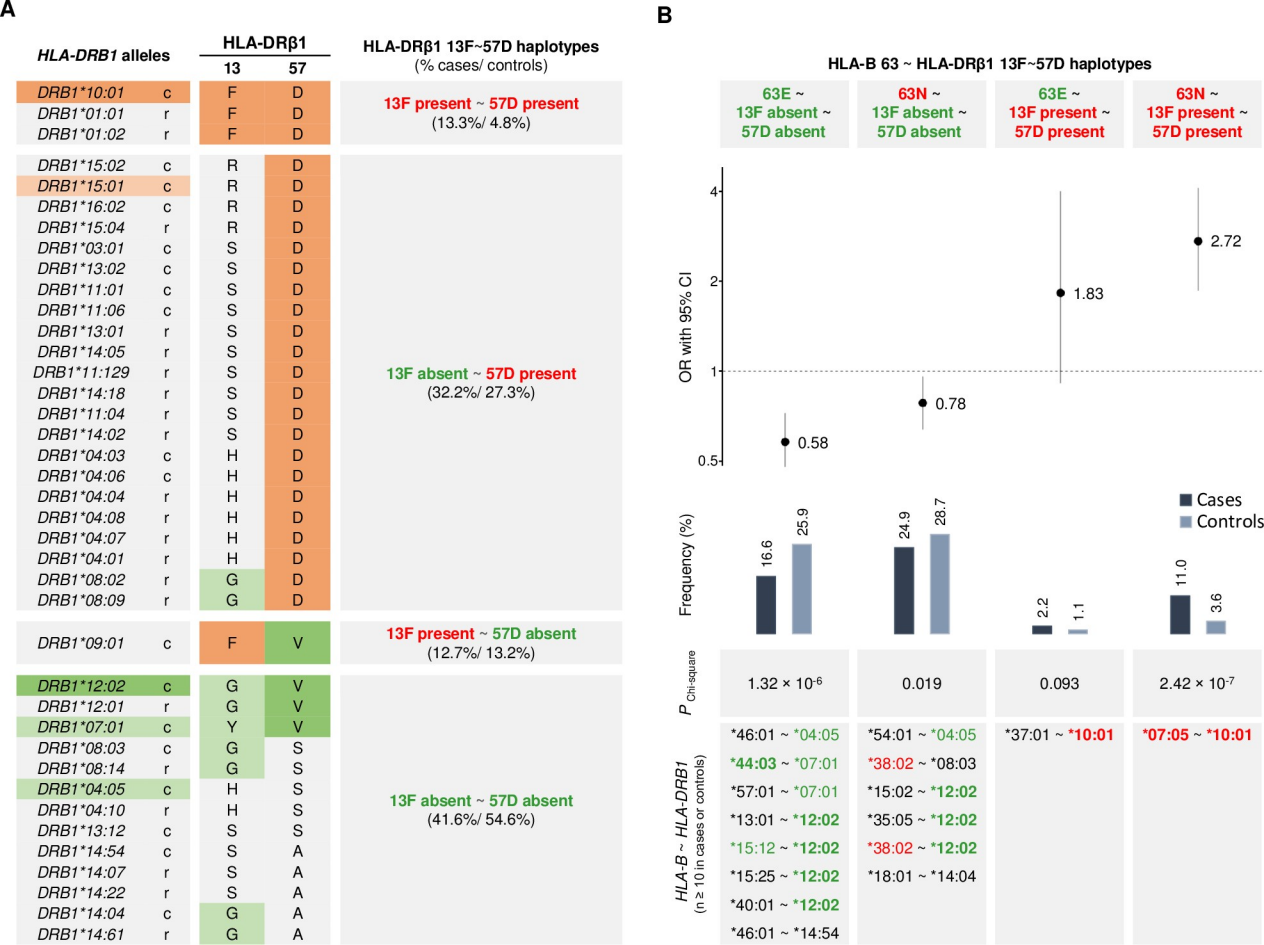
HLA-DRβ1 13F~ 57D~ HLA-B 63N/E haplotypes correlated with the associated *HLA-DRB1* and *HLA-B* alleles. **A)** Distribution of phased haplotypes of the risk amino acids HLA-DRβ1 13F and 57D among *HLA-DRB1* alleles. The left column presents the 39 four-digit *HLA-DRB1* alleles genotyped in the present Vietnamese sample. Rare (< 1%) and common (≥ 1%) alleles are indicated by r and c, respectively. The middle column presents the amino acid residues at HLA-DRβ1 positions 13 and 57 for each HLA allele. In the left and middle columns, the HLA alleles and amino acids with *P* < 0.01 are colored in orange or green if their presence corresponded to risk or protection from leprosy, respectively (S1 and S4 Tables). Markers with multiple-testing significance in the univariable analysis are indicated by a darker orange/green. In the right column, HLA alleles are grouped based on the haplotype of the presence/absence of HLA-DRβ1 13F and 57D. **B)** Contribution of the HLA-B 63N/E biallelic amino acid polymorphism to the association of HLA-DRβ1 13F present~ 57D present and HLA-DRβ1 13F absent ~ 57D absent with leprosy. The results of the four estimated phased haplotypes are presented by columns. Odds ratios (OR), 95% confidence intervals (95% CI) and *P*_Chi-square_ were determined for the four haplotypes using the pool of remaining haplotypes as reference (44.7% in cases and 40.4% in controls), except haplotypes with missing data (0.7% in cases and 0.3% in controls). Four-digit *HLA-B*~ *HLA-DRB1* haplotypes belonging to each group and presenting counts higher or equal to ten chromosomes in cases and/or controls are listed at the bottom. HLA alleles and single amino acids that as binary markers presented *P* < 0.01 in the genotypic univariable analysis are colored in red and green when associated with risk or protection from leprosy, respectively. Markers that reached the multiple-testing thresholds are indicated in bold (S1 and S4 Tables).

To better understand how the HLA-DRβ1 amino acid residues contributed to the association of the *HLA-DRB1* alleles, we analyzed the phased haplotype between HLA-DRβ1 13F and 57D based on the protein sequence of the four-digit HLA alleles (Fig 3A). HLA-DRβ1 position 13 and 57 were multi-allelic in the studied population with five and three additional amino acids besides 13F and 57D, respectively. Interestingly, the haplotype of the two risk residues was only present on the risk allele HLA-DRB1*10:*01* and two rare alleles, HLA-DRB1*01:*01* and HLA-DRB1*01:02 (HLA-DRβ1 13F present ~ 57D present haplotype in Fig 3A). Due to their low frequency, the HLA-DRB1*01 alleles were not included in the association analyses (see Methods). However, we noted that the HLA-DRB1*01 group was more frequent in leprosy cases than in healthy controls (frequency of 0.7% and 0.3% respectively). On the other hand, both risk amino acids were absent in 13 *HLA-DRB1* alleles including the protective HLA-DRB1*12 (HLA-DRβ1 13F absent ~ 57D absent haplotype in Fig 3A). This suggested the haplotypes HLA-DRβ1 13F present ~ 57D present and HLA-DRβ1 13F absent ~ 57D absent as main causes of the risk and protective effects of leprosy associated *HLA-DRB1* alleles.

In the HLA-B protein, position 63 was biallelic in our dataset with the risk asparagine (HLA-B 63N) as the major allele among cases and the protective glutamic acid (HLA-B 63E) as the major allele among controls (HLA-B 63E had frequencies of 46.3% in cases and 52.1% in controls). We analyzed the haplotypic effect between the amino acid at HLA-B position 63 with the major risk HLA-DRβ1 13F present ~ 57D present and the major protective HLA-DRβ1 13F absent~ 57D absent haplotypes. For that, we estimated the phased haplotype between *HLA-B* and *HLA-DRB1*. We observed that the strength of association of the protective HLA-DRβ1 13F absent~ 57D absent was increased by the presence of the protective HLA-B 63E on the same haplotype, while HLA-DRβ1 13F present ~ 57D present had a stronger risk effect when on the same haplotype with HLA-B 63N (Fig 3B).

HLA-B*07:*05* and HLA-DRB1*10:*01*, both from signal #1 in the HLA allele analysis (Fig 1), belonged to the risk combination HLA-B 63N ~ HLA-DRβ1 13F present ~ 57D present and were the majority of samples in this group (Fig 3B). The protective HLA-B*44:*03* from signal #2 (Fig 1) was mostly found on the background of HLA-DRB1*07:*01* haplotype in the Vietnamese population (S1 Fig) and HLA-B*44:03~ HLA-DRB1*07:*01* haplotype belonged to the protective HLA-B 63E ~ HLA-DRβ1 13F absent~ 57D absent group (Fig 3B). Interestingly, HLA-DRB1*07:*01* presented a trend toward association as a protective allele in the univariable analysis (OR = 0.59, *P* = 2.83 × 10^−3^). When we analyzed HLA-B*44:*03* and HLA-DRB1*07:*01* by reciprocal conditional analysis we found that the HLA-DRB1*07:*01* suggestive signal was dependent on the significant HLA-B*44:*03* allele (S3 Table) and was included in signal #2 (Fig 1). Finally, haplotypes including the protective HLA-DRB1*12:*02* allele from signal #3 in the protective HLA-B 63E ~ HLA-DRβ1 13F absent~ 57D absent haplotype group consisted of different less common and non-significant *HLA-B* alleles with glutamic acid at position 63 (Fig 3B). These results indicated that the class I HLA-B amino acid change contributed to the association signal of the class II variants and illustrates how protective and susceptibility effects of classical HLA alleles are dependent on the presence/absence of specific amino acids.

## Discussion

The MHC region has been consistently identified as chromosomal location of strong genetic leprosy risk factors, including in our recent Vietnamese GWAS where three independent association signals were identified in the MHC region [7]. While genetic studies have implicated non-classical MHC genes like *LTA* and *MICA* in leprosy susceptibility, the main effects have been assigned to classical class I and class II HLA genes [11, 35]. Employing candidate gene approaches, these genes have long been the object of genetic studies of leprosy, mainly trying to identify clinical subtype specific effects (rev in [9]). However, the extreme polymorphic nature of class I and class II HLA genes combined with extensive, population-specific LD have provided a major challenge for the replication of reported allele associations across studies. HLA imputation using GWAS data has been demonstrated to be an effective method for MHC fine mapping. However, this approach also presents some limitations. Imputation accuracy of low frequency HLA alleles is dependent on the similarity of the ethnicities between the pre-built reference panel and the studied population sample since alleles not present in the panel cannot be imputed. HLA imputation is limited to the genes present in the reference panel. Moreover, HLA allele resolution of the imputed data is defined by the resolution of the HLA typing method used to generate the reference panel. Hence, in the present study, we elected to perform high-resolution HLA typing by NGS to obtain accurate HLA genotypes (low ambiguity rate) independently to our GWAS data and pre-build reference panels. The use of NGS to genotype classical HLA genes in the Vietnamese population allowed us to gain a comprehensive understanding of the alleles implicated in leprosy susceptibility. Based on HLA allele analysis, we detected independent signals of association with risk and protection from leprosy of class I and class II alleles (Fig 1).

The strongest leprosy susceptibility factors in our study were the HLA-DRB1*10:*01* and HLA-DQA1*01:*05* alleles which are in complete LD in the Vietnamese population. These two alleles are tagging an extended haplotype which spans from *HLA-A* to *HLA-DPB1*, a distance of approximately 3.15 Mb (signal #1 in Fig 1). These findings are strongly supported by a study of three Indian leprosy cohorts and our family-based leprosy GWAS in Vietnam [5, 7]. In the Indian population samples, the authors reported a strong risk effect for leprosy of MHC class II SNP rs1071630 in two case-control samples from New Delhi and Kolkata [5]. A significant but weaker effect was seen in a family-based population sample from Southern India for the same SNP [5]. In the Vietnamese leprosy GWAS, SNP rs3187964 was identified as the most significant independent signal in the MHC region [7]. Employing a subset of imputed data, we found that both rs1071630 and rs3187964 were in complete LD with HLA-DQA1*01 in our sample (*r*^*2*^ = 1 and *r*^*2*^ = 0.98 respectively). HLA-DQA1*01 was significantly associated with leprosy and presented frequencies of 43.1% and 32.8% in cases and controls, respectively (OR = 1.56, *P* = 8.34 × 10^−7^, Table 2). HLA-DQA1*01 is composed of five four-digit alleles that share specific amino acid residues (*01:01 to *01:05), including the major risk allele HLA-DQA1*01:*05* (Table 2). Conditioning on HLA-DQA1*01 did not abolish the association of HLA-DQA1*01:*05* (*P*_conditional_ = 1.25 × 10^−6^). Conversely, conditioning on HLA-DQA1*01:*05* accounted for most of the signal of HLA-DQA1*01 (*P*_conditional_ = 0.0103). Together, these data showed that rs1071630 and rs3187964 are tag SNPs for the major risk signal in our sample.

Similarly, we had shown SNP rs2394885 to be a major leprosy risk factor in two large samples from Vietnam and India [12]. We had also shown that this SNP tags the HLA-C*15:*05* allele. Here, we replicated this previous observation (r^2^ = 0.63) and found the same SNP also to be in strong LD with HLA-B*07:*05* (r^2^ = 0.63) and HLA-A*29:*01* (r^2^ = 0.58). In the Vietnamese leprosy GWAS, we found that rs2394885 belonged to a large bin of SNPs in strong LD that included the most significant independent class I region SNP rs114598080 of the GWAS [7]. All three tagged class I alleles (HLA-C*15:*05*, HLA-B*07:*05*, HLA-A*29:*01*) are part of signal #1 in our study (Fig 1). Taken together the combined results of the present and previous studies strongly support the role of HLA-DRB1*10:*01*~ HLA-DQA1*01:*05* as major leprosy risk factor in independent Indian and Vietnamese samples.

Previous studies in large, well powered samples of Chinese leprosy patients had identified HLA-DRB1*15:*01* as the strongest leprosy associated HLA allele (OR = 2.17, *P* = 4.21 × 10^−44^) [3, 4]. These studies had not detected a significant risk effect of the HLA-DRB1*10:*01* and HLA-DQA1*01:*05* alleles. In our sample, we detected a risk effect of HLA-DRB1*15:*01* with similar OR to the one reported for the Chinese patients which failed to pass correction for multiple testing (OR = 2.02, *P* = 1.11 × 10^−3^). The reduced significance of HLA-DRB1*15:*01* in the Vietnamese patients is most likely a result of the substantially lower allele frequency in our sample (6.5% and 3.2% in cases and controls) as compared to the Chinese sample (11.7%) [3]. Accounting for the signals tagged by HLA-DQA1*01:*05*, HLA-C*07:*06* and HLA-DRB1*12 did not abolish the borderline effect for the HLA-DRB1*15:*01* allele (*P*_conditional_ = 1.86 × 10^−3^, S5 Table). Hence, HLA-DRB1*15:*01* was indeed an independent leprosy risk factor for the Vietnamese patients which was also supported by the observation that HLA-DRB1*15:*01* contributed significantly to leprosy risk independently of HLA-DRB1*10:*01*~ HLA-DQA1*01:*05*. Previously, two small studies reported a leprosy *per se* risk effect of HLA-DRB1*15:*01* and a serotype linked to HLA-DQA1*01, and a well powered study of Brazilian leprosy patients detected a risk effect for both HLA-DRB1*10 and HLA-DRB1*15 [36–38]. Taken together, these results implicated HLA-DRB1*10:01/ HLA-DQA1*01:*05* and HLA-DRB1*15:*01* as strong global leprosy risk factors.

The strongest protective signal was associated with the HLA-C*07:*06*/HLA-B*44:*03* alleles. The two alleles were mostly found on a haplotype with HLA-DRB1*07:*01* (D’ = 0.77), and collectively made up signal #2 in our study (Fig 1 and S1 Fig). The univariable borderline association of HLA-DRB1*07:*01* with leprosy was fully explained by HLA-C*07:06/HLA-B*44:*03* by multivariable analysis. Hence, signal #2 is consistent with previous results in a large case-control sample from Brazil that did not test class I alleles but detected HLA-DRB1*07 as protective factor [38]. The second resistance signal (signal #3) in our study was associated with allele HLA-DRB1*12:*02*. This finding was consistent with the reported protective effect of HLA-DRB1*12 in Brazilian and Indonesian leprosy patients [38, 39]. In large studies in Chinese patients, the strongest protective effect was assigned to HLA-DQB1*04:*01* [3]. In both the Vietnamese and the Chinese populations, HLA-DQB1*04:*01* is part of a haplotype which includes HLA-DQA1*03:*03* and HLA-DRB1*04:*05* (S1 Fig), and in a meta-analysis of studies in Chinese patients HLA-DQA1*03:*03* was identified as main leprosy resistance factor [10]. While in our study both HLA-DQA1*03:*03* and HLA-DRB1*04:*05* did not pass the significance threshold required by multiple testing, we did observe a strong protective effect for both alleles independently of the three significant HLA allele signals (OR_conditional_ = 0.50, *P*_conditional_ = 2.65 × 10^−3^ and OR_conditional_ = 0.49, *P*_conditional_ = 3.02 × 10^−3^, respectively) that can be considered as replication of the Chinese studies (S5 Table). Hence, while significance of the protective effect differed among Chinese and Vietnamese patients the effect sizes obtained in Vietnamese patients were consistent with those obtained for Chinese patients.

The leprosy protective signal #2 is dominated by alleles that are part of a HLA-C*07:*06* ~ HLA-B*44:03 ~ HLA-DRB1*07:*01* haplotype (Fig 1). Of note, HLA-DRB1*07:*01* had previously been implicated in two main inflammatory bowel diseases: Crohn’s disease (CD) and ulcerative colitis (UC). Interestingly, HLA-DRB1*07:*01* was protective for UC while it was a risk marker for CD [40]. A meta-analysis of *HLA-DRB1* alleles and risk of tuberculosis (TB) in Asian patients found HLA-DRB1*07:*01* associated with protection from TB [41]. The same meta-analysis also identified HLA-DRB1*12:*02* (protective signal #3 in Fig 1) as TB protective marker with borderline significance [41]. In addition, HLA-DRB1*12 and *12:*02* had also been associated with protection from periodontal oral infections and recurrent typhoid fever [42, 43]. An allele that needs to be considered is HLA-DRB1*04:*05* which was associated with protection from leprosy in the present as well as other studies [38, 44, 45]. Previously, HLA-DRB1*04:*05* had been found protective for enteric fever in Vietnamese patients [46]. In addition, HLA-DRB1*04:*05* had been reported as associated with protection from UC and risk for CD in the Japanese population [47]. Finally, the leprosy risk allele HLA-DRB1*15:*01* was a major risk factor for multiple sclerosis in earlier studies and has been associated with risk for UC and Parkinson’s disease [40, 48–50]. The combined results showed that HLA alleles implicated in leprosy susceptibility have a wider impact on infectious and inflammatory diseases. Importantly, these results also further support the genetic overlap between leprosy, inflammatory bowel disease and Parkinson’s disease [1, 16, 51].

Our study demonstrated the challenges posed by the very strong LD across the HLA complex. While independent risk and protective signals could be defined, it was in general not possible by genetic means to implicate a single class I/II allele as cause of the observed associations. To disentangle the complex pattern of correlated alleles, we investigated the association of single polymorphic amino acids within class I and class II proteins. We identified four amino acids in HLA-DRβ1 (phenylalanine and aspartic acid in positions 13 and 57), HLA-B (glutamic acid in position 63), and HLA-A (lysine in position 19) that fully explained the associations of class I and class II alleles with leprosy in our sample (S5 and S6 Tables). Model selection metrics, such as AIC, have been useful to identify the combination of independent HLA variants that represent the best-fitting model for the data in previous studies [52, 53]. Hence, we used AIC to compare the allele-based and amino acid-based models in our dataset. We showed that the model based on only four amino acids better explained the HLA effect on leprosy than the model based on HLA alleles that tag a number of highly correlated alleles (Table 3). Three of the four amino acids are located within the peptide-binding groove of the HLA-DRβ1 and HLA-B proteins (S2 Fig). Amino acid changes in the peptide-binding groove can impact on the HLA molecule interaction with the antigen peptide. In the HLA-DR molecule, amino acid changes at the two HLA-DRβ1 polymorphic positions 57 and 13 are predicted to impact on the peptide-binding repertoire size [54]. Moreover, aspartic acid at position 57 of HLA-DRβ1 interacts with a conserved residue in HLA-DRα and this interaction appears lost in the absence of aspartic acid [55]. Interestingly, this HLA-DR interaction is the same as in HLA-DQ for the well-known Type 1 diabetes-protective HLA-DQβ1 57D [56–58]. In HLA-B, an *in silico* analysis has indicated that a single substitution in position 63 of the protein can have a strong impact on the protein repertoire that bind the molecule, suggesting this position as crucial for peptide binding [59]. Taken together, these findings suggest that the strong impact on leprosy susceptibility found in our study can be traced to changes in the HLA molecule structure as well as to specific HLA molecule-antigen peptide interactions. Additional studies will be required to fully comprehend the exact range of HLA-*M*. *leprae* antigen interactions as well as to investigate the non-additive and interactive risk effect of HLA variants in leprosy. Functional studies analyzing the impact of HLA-DRβ1 57D and 13F, HLA-B 63E and HLA-A 19K in the context of the specific protein structure of the associated HLA alleles will provide additional insight in the role of HLA molecules in leprosy susceptibility. Given the ongoing efforts of developing a leprosy vaccine, it will be useful to assure that vaccine antigens can overcome this specific genetic restriction of leprosy susceptibility.

## Supporting information

S1 Fig Haplotype structure of the four-digit alleles for class I *HLA-A*, *-C* and *-B* and class II *HLA-DRB1*, *-DQA1*, *-DQB1* and *-DPB1* in the Vietnamese sample. The two panels show the HLA haplotype structure in **A**) healthy controls (N = 468) and **B**) leprosy cases (N = 687). The columns represent observed alleles of seven HLA class I and class II genes, where each box corresponds to a specific four-digit HLA allele. The height of the box is relative to the observed HLA allele frequency. Alleles associated with risk or protection from leprosy *per se* are shown in black and green boxes, respectively (see Fig 1). Each grey line connects two HLA alleles in consecutives HLA genes, where the thickness of the line is based on the frequency of the haplotype. Haplotypes between associated alleles in consecutives genes are highlighted in darker grey.
(TIF)

S2 Fig HLA-DRβ1 positions 13 and 57 and HLA-B position 63, but not HLA-A position 19, are located in the peptide-binding grooves of the respective HLA proteins. Three-dimensional ribbon representation of **A**) HLA-DR, **B**) HLA-B and **C**) HLA-A peptide binding grooves. HLA-DRα, HLA-DRβ1, HLA-B, HLA-A and microglobulin are shown in green, blue, pink, purple and grey, respectively. HLA-DRβ1 positions 13 and 57 (**A**), HLA-B position 63 (**B**) and HLA-A position 19 (**C**) are shown as spheres and highlighted in red.
(TIF)

S1 Table Association analysis of leprosy *per se*, leprosy subtype polarization or type-1 reaction phenotypes against HLA alleles binary markers from 11 classic HLA genes. (XLSX)

S2 Table Forward conditional analysis of HLA alleles that reached study-wide significant association with leprosy per se. (XLSX)

S3 Table Linkage disequilibrium analysis in healthy controls and pairwise reciprocal conditional analysis of leprosy-associated HLA alleles. (XLSX)

S4 Table Association analysis of leprosy *per se* and HLA amino acids in HLA-A, HLA-C, HLA-B, HLA-DRβ1 and HLA-DQα1. (XLSX)

S5 Table HLA alleles with conditional *P*-value lower than 0.01 after adjusting on HLA-DQA1*01:05, HLA-C*07:06 and HLA-DRB1*12. (XLSX)

S6 Table Impact on HLA alleles association with leprosy *per se* by conditioning the logistic regression on HLA-DRβ1 57D, HLA-DRβ1 13F and HLA-B 63E in a forward fashion. (XLSX)

S1 Data Genotypes of the 622 tested HLA alleles and amino acid binary markers in 468 controls and 687 leprosy cases (coded as 1 and 2, respectively). Genotypes are provided in PLINK binary format (bed/bim/fam files) from PLINK v1.9 (http://www.cog-genomics.org/plink/1.9/).
(GZ) Click here for additional data file.

## References

[1] Fava VM, Dallmann-Sauer M, Schurr E. Genetics of leprosy: today and beyond. Hum Genet. 2019 Epub 2019/11/13. 10.1007/s00439-019-02087-5 . 31713021

[2] Zhang FR, Huang W, Chen SM, Sun LD, Liu H, Li Y, et al Genomewide association study of leprosy. N Engl J Med. 2009;361(27):2609–18. Epub 2009/12/19. 10.1056/NEJMoa0903753 . 20018961

[3] Liu H, Irwanto A, Fu X, Yu G, Yu Y, Sun Y, et al Discovery of six new susceptibility loci and analysis of pleiotropic effects in leprosy. Nat Genet. 2015;47(3):267–71. Epub 2015/02/03. 10.1038/ng.3212 . 25642632

[4] Wang Z, Sun Y, Fu X, Yu G, Wang C, Bao F, et al A large-scale genome-wide association and meta-analysis identified four novel susceptibility loci for leprosy. Nat Commun. 2016;7:13760 Epub 2016/12/16. 10.1038/ncomms13760 27976721 PMC5172377

[5] Wong SH, Gochhait S, Malhotra D, Pettersson FH, Teo YY, Khor CC, et al Leprosy and the adaptation of human toll-like receptor 1. PLoS Pathog. 2010;6:e1000979 Epub 2010/07/10. 10.1371/journal.ppat.1000979 20617178 PMC2895660

[6] Liu H, Wang Z, Li Y, Yu G, Fu X, Wang C, et al Genome-Wide Analysis of Protein-Coding Variants in Leprosy. J Invest Dermatol. 2017;137(12):2544–51. Epub 2017/08/27. 10.1016/j.jid.2017.08.004 . 28842327

[7] Gzara C, Dallmann-Sauer M, Orlova M, Van Thuc N, Thai VH, Fava VM, et al Family-based genome-wide association study of leprosy in Vietnam. PLoS Pathog. 2020;16(5):e1008565 Epub 2020/05/19. 10.1371/journal.ppat.1008565 32421744 PMC7259797

[8] Trowsdale J, Knight JC. Major histocompatibility complex genomics and human disease. Annu Rev Genomics Hum Genet. 2013;14:301–23. Epub 2013/07/24. 10.1146/annurev-genom-091212-153455 23875801 PMC4426292

[9] Jarduli LR, Sell AM, Reis PG, Sippert EA, Ayo CM, Mazini PS, et al Role of HLA, KIR, MICA, and cytokines genes in leprosy. Biomed Res Int. 2013;2013:989837 Epub 2013/08/13. 10.1155/2013/989837 23936864 PMC3722889

[10] Zhang X, Cheng Y, Zhang Q, Wang X, Lin Y, Yang C, et al Meta-Analysis Identifies Major Histocompatiblity Complex Loci in or Near HLA-DRB1, HLA-DQA1, HLA-C as Associated with Leprosy in Chinese Han Population. J Invest Dermatol. 2019;139(4):957–60. Epub 2018/11/06. 10.1016/j.jid.2018.09.029 . 30389493

[11] Alcais A, Alter A, Antoni G, Orlova M, Nguyen VT, Singh M, et al Stepwise replication identifies a low-producing lymphotoxin-alpha allele as a major risk factor for early-onset leprosy. Nat Genet. 2007;39(4):517–22. Epub 2007/03/14. 10.1038/ng2000 . 17353895

[12] Alter A, Huong NT, Singh M, Orlova M, Van Thuc N, Katoch K, et al Human leukocyte antigen class I region single-nucleotide polymorphisms are associated with leprosy susceptibility in Vietnam and India. J Infect Dis. 2011;203(9):1274–81. Epub 2011/04/05. 10.1093/infdis/jir024 21459816 PMC3069725

[13] Fava VM, Manry J, Cobat A, Orlova M, Van Thuc N, Moraes MO, et al A genome wide association study identifies a lncRna as risk factor for pathological inflammatory responses in leprosy. PLoS Genet. 2017;13(2):e1006637 Epub 2017/02/22. 10.1371/journal.pgen.1006637 28222097 PMC5340414

[14] Grant AV, Alter A, Huong NT, Orlova M, Van Thuc N, Ba NN, et al Crohn's disease susceptibility genes are associated with leprosy in the Vietnamese population. J Infect Dis. 2012;206(11):1763–7. Epub 2012/09/18. 10.1093/infdis/jis588 . 22984114

[15] Gaschignard J, Grant AV, Thuc NV, Orlova M, Cobat A, Huong NT, et al Pauci- and Multibacillary Leprosy: Two Distinct, Genetically Neglected Diseases. PLoS Negl Trop Dis. 2016;10(5):e0004345 Epub 2016/05/25. 10.1371/journal.pntd.0004345 27219008 PMC4878860

[16] Fava VM, Xu YZ, Lettre G, Van Thuc N, Orlova M, Thai VH, et al Pleiotropic effects for Parkin and LRRK2 in leprosy type-1 reactions and Parkinson's disease. Proc Natl Acad Sci U S A. 2019;116(31):15616–24. Epub 2019/07/17. 10.1073/pnas.1901805116 31308240 PMC6681704

[17] Duke JL, Lind C, Mackiewicz K, Ferriola D, Papazoglou A, Gasiewski A, et al Determining performance characteristics of an NGS-based HLA typing method for clinical applications. HLA. 2016;87(3):141–52. Epub 2016/02/18. 10.1111/tan.12736 . 26880737

[18] Omixon Biocomputing Ltd. Omixon HLA Twin RUO—2.1.4: Handbook. Budapest, Hungary.

[19] Robinson J, Barker DJ, Georgiou X, Cooper MA, Flicek P, Marsh SGE. IPD-IMGT/HLA Database. Nucleic Acids Res. 2020;48(D1):D948–D55. Epub 2019/11/02. 10.1093/nar/gkz950 31667505 PMC7145640

[20] Omixon Biocomputing Ltd. Omixon HLA Twin RUO—2.5.0: User guide. Budapest, Hungary.

[21] Jia X, Han B, Onengut-Gumuscu S, Chen WM, Concannon PJ, Rich SS, et al Imputing amino acid polymorphisms in human leukocyte antigens. PLoS One. 2013;8(6):e64683 Epub 2013/06/14. 10.1371/journal.pone.0064683 23762245 PMC3675122

[22] R Core Team. R: A language and environment for statistical computing. R Foundation for Statistical Computing, Vienna, Austria 2020 Available from: https://www.R-project.org/.

[23] Li MX, Yeung JM, Cherny SS, Sham PC. Evaluating the effective numbers of independent tests and significant p-value thresholds in commercial genotyping arrays and public imputation reference datasets. Hum Genet. 2012;131(5):747–56. Epub 2011/12/07. 10.1007/s00439-011-1118-2 22143225 PMC3325408

[24] Chang CC, Chow CC, Tellier LC, Vattikuti S, Purcell SM, Lee JJ. Second-generation PLINK: rising to the challenge of larger and richer datasets. Gigascience. 2015;4:7 Epub 2015/02/28. 10.1186/s13742-015-0047-8 25722852 PMC4342193

[25] Akaike H. A new look at the statistical model identification. IEEE Transactions on Automatic Control. 1974;19(6):716–23. 10.1109/tac.1974.1100705

[26] Burnham K, Anderson D. Model selection and multimodel inference: a practical information-theoretic approach. 2nd ed. New York: Springer-Verlag; 2002.

[27] Browning BL, Browning SR. A unified approach to genotype imputation and haplotype-phase inference for large data sets of trios and unrelated individuals. Am J Hum Genet. 2009;84(2):210–23. Epub 2009/02/10. 10.1016/j.ajhg.2009.01.005 19200528 PMC2668004

[28] Aragon TJ. epitools: Epidemiology Tools. R package version 0.5–10. 2017. Available from: https://CRAN.R-project.org/package=epitools.

[29] Kumasaka N, Nakamura Y, Kamatani N. The textile plot: a new linkage disequilibrium display of multiple-single nucleotide polymorphism genotype data. PLoS One. 2010;5(4):e10207 Epub 2010/05/04. 10.1371/journal.pone.0010207 20436909 PMC2860502

[30] Okada Y, Momozawa Y, Ashikawa K, Kanai M, Matsuda K, Kamatani Y, et al Construction of a population-specific HLA imputation reference panel and its application to Graves' disease risk in Japanese. Nat Genet. 2015;47(7):798–802. Epub 2015/06/02. 10.1038/ng.3310 . 26029868

[31] Pettersen EF, Goddard TD, Huang CC, Couch GS, Greenblatt DM, Meng EC, et al UCSF Chimera—a visualization system for exploratory research and analysis. J Comput Chem. 2004;25(13):1605–12. Epub 2004/07/21. 10.1002/jcc.20084 . 15264254

[32] Gunther S, Schlundt A, Sticht J, Roske Y, Heinemann U, Wiesmuller KH, et al Bidirectional binding of invariant chain peptides to an MHC class II molecule. Proc Natl Acad Sci U S A. 2010;107(51):22219–24. Epub 2010/12/01. 10.1073/pnas.1014708107 21115828 PMC3009805

[33] Zernich D, Purcell AW, Macdonald WA, Kjer-Nielsen L, Ely LK, Laham N, et al Natural HLA class I polymorphism controls the pathway of antigen presentation and susceptibility to viral evasion. J Exp Med. 2004;200(1):13–24. Epub 2004/07/01. 10.1084/jem.20031680 15226359 PMC2213310

[34] Bulek AM, Cole DK, Skowera A, Dolton G, Gras S, Madura F, et al Structural basis for the killing of human beta cells by CD8(+) T cells in type 1 diabetes. Nat Immunol. 2012;13(3):283–9. Epub 2012/01/17. 10.1038/ni.2206 22245737 PMC3378510

[35] Tosh K, Ravikumar M, Bell JT, Meisner S, Hill AV, Pitchappan R. Variation in MICA and MICB genes and enhanced susceptibility to paucibacillary leprosy in South India. Hum Mol Genet. 2006;15(19):2880–7. Epub 2006/08/23. 10.1093/hmg/ddl229 . 16923796

[36] Rani R, Fernandez-Vina MA, Zaheer SA, Beena KR, Stastny P. Study of HLA class II alleles by PCR oligotyping in leprosy patients from north India. Tissue Antigens. 1993;42(3):133–7. Epub 1993/09/01. 10.1111/j.1399-0039.1993.tb02179.x . 8284786

[37] Schauf V, Ryan S, Scollard D, Jonasson O, Brown A, Nelson K, et al Leprosy associated with HLA-DR2 and DQw1 in the population of northern Thailand. Tissue Antigens. 1985;26(4):243–7. Epub 1985/10/01. 10.1111/j.1399-0039.1985.tb00966.x . 3878012

[38] Vanderborght PR, Pacheco AG, Moraes ME, Antoni G, Romero M, Verville A, et al HLA-DRB1*04 and DRB1*10 are associated with resistance and susceptibility, respectively, in Brazilian and Vietnamese leprosy patients. Genes Immun. 2007;8(4):320–4. Epub 2007/03/31. 10.1038/sj.gene.6364390 . 17396103

[39] Soebono H, Giphart MJ, Schreuder GM, Klatser PR, de Vries RR. Associations between HLA-DRB1 alleles and leprosy in an Indonesian population. Int J Lepr Other Mycobact Dis. 1997;65(2):190–6. Epub 1997/06/01. . 9251590

[40] Goyette P, Boucher G, Mallon D, Ellinghaus E, Jostins L, Huang H, et al High-density mapping of the MHC identifies a shared role for HLA-DRB1*01:03 in inflammatory bowel diseases and heterozygous advantage in ulcerative colitis. Nat Genet. 2015;47(2):172–9. Epub 2015/01/07. 10.1038/ng.3176 25559196 PMC4310771

[41] Li CP, Zhou Y, Xiang X, Zhou Y, He M. Relationship of HLA-DRB1 gene polymorphism with susceptibility to pulmonary tuberculosis: updated meta-analysis. Int J Tuberc Lung Dis. 2015;19(7):841–9. Epub 2015/06/10. 10.5588/ijtld.14.0521 . 26056112

[42] Mauramo M, Ramseier AM, Buser A, Tiercy JM, Weiger R, Waltimo T. Associations of HLA-A, -B and -DRB1 types with oral diseases in Swiss adults. PLoS One. 2014;9(7):e103527 Epub 2014/07/30. 10.1371/journal.pone.0103527 25072155 PMC4114782

[43] Dharmana E, Joosten I, Tijssen HJ, Gasem MH, Indarwidayati R, Keuter M, et al HLA-DRB1*12 is associated with protection against complicated typhoid fever, independent of tumour necrosis factor alpha. Eur J Immunogenet. 2002;29(4):297–300. Epub 2002/07/18. 10.1046/j.1365-2370.2002.00318.x . 12121274

[44] Joko S, Numaga J, Kawashima H, Namisato M, Maeda H. Human leukocyte antigens in forms of leprosy among Japanese patients. Int J Lepr Other Mycobact Dis. 2000;68(1):49–56. Epub 2000/06/02. . 10834069

[45] Hsieh NK, Chu CC, Lee NS, Lee HL, Lin M. Association of HLA-DRB1*0405 with resistance to multibacillary leprosy in Taiwanese. Hum Immunol. 2010;71(7):712–6. Epub 2010/04/01. 10.1016/j.humimm.2010.03.007 . 20353806

[46] Dunstan SJ, Hue NT, Han B, Li Z, Tram TT, Sim KS, et al Variation at HLA-DRB1 is associated with resistance to enteric fever. Nat Genet. 2014;46(12):1333–6. Epub 2014/11/11. 10.1038/ng.3143 25383971 PMC5099079

[47] Arimura Y, Isshiki H, Onodera K, Nagaishi K, Yamashita K, Sonoda T, et al Characteristics of Japanese inflammatory bowel disease susceptibility loci. Journal of Gastroenterology. 2013;49(8):1217–30. Epub 2013/08/15. 10.1007/s00535-013-0866-2 . 23942620

[48] Patsopoulos NA, Barcellos LF, Hintzen RQ, Schaefer C, van Duijn CM, Noble JA, et al Fine-mapping the genetic association of the major histocompatibility complex in multiple sclerosis: HLA and non-HLA effects. PLoS Genet. 2013;9(11):e1003926 Epub 2013/11/28. 10.1371/journal.pgen.1003926 24278027 PMC3836799

[49] Wissemann WT, Hill-Burns EM, Zabetian CP, Factor SA, Patsopoulos N, Hoglund B, et al Association of Parkinson disease with structural and regulatory variants in the HLA region. Am J Hum Genet. 2013;93(5):984–93. Epub 2013/11/05. 10.1016/j.ajhg.2013.10.009 24183452 PMC3824116

[50] Sulzer D, Alcalay RN, Garretti F, Cote L, Kanter E, Agin-Liebes J, et al T cells from patients with Parkinson's disease recognize alpha-synuclein peptides. Nature. 2017;546(7660):656–61. Epub 2017/06/22. 10.1038/nature22815 28636593 PMC5626019

[51] Hui KY, Fernandez-Hernandez H, Hu J, Schaffner A, Pankratz N, Hsu NY, et al Functional variants in the LRRK2 gene confer shared effects on risk for Crohn's disease and Parkinson's disease. Sci Transl Med. 2018;10(423):eaai7795 Epub 2018/01/13. 10.1126/scitranslmed.aai7795 29321258 PMC6028002

[52] Morris DL, Taylor KE, Fernando MM, Nititham J, Alarcon-Riquelme ME, Barcellos LF, et al Unraveling multiple MHC gene associations with systemic lupus erythematosus: model choice indicates a role for HLA alleles and non-HLA genes in Europeans. Am J Hum Genet. 2012;91(5):778–93. Epub 2012/10/23. 10.1016/j.ajhg.2012.08.026 23084292 PMC3487133

[53] Ferreiro-Iglesias A, Lesseur C, McKay J, Hung RJ, Han Y, Zong X, et al Fine mapping of MHC region in lung cancer highlights independent susceptibility loci by ethnicity. Nat Commun. 2018;9(1):3927 Epub 2018/09/27. 10.1038/s41467-018-05890-2 30254314 PMC6156406

[54] Manczinger M, Boross G, Kemeny L, Muller V, Lenz TL, Papp B, et al Pathogen diversity drives the evolution of generalist MHC-II alleles in human populations. PLoS Biol. 2019;17(1):e3000131 Epub 2019/02/01. 10.1371/journal.pbio.3000131 30703088 PMC6372212

[55] Brown JH, Jardetzky TS, Gorga JC, Stern LJ, Urban RG, Strominger JL, et al Three-dimensional structure of the human class II histocompatibility antigen HLA-DR1. Nature. 1993;364(6432):33–9. Epub 1993/07/01. 10.1038/364033a0 . 8316295

[56] Todd JA, Bell JI, McDevitt HO. HLA-DQ beta gene contributes to susceptibility and resistance to insulin-dependent diabetes mellitus. Nature. 1987;329(6140):599–604. Epub 1987/10/15. 10.1038/329599a0 . 3309680

[57] Hu X, Deutsch AJ, Lenz TL, Onengut-Gumuscu S, Han B, Chen WM, et al Additive and interaction effects at three amino acid positions in HLA-DQ and HLA-DR molecules drive type 1 diabetes risk. Nat Genet. 2015;47(8):898–905. Epub 2015/07/15. 10.1038/ng.3353 26168013 PMC4930791

[58] Gerasimou P, Nicolaidou V, Skordis N, Picolos M, Monos D, Costeas PA. Combined effect of glutamine at position 70 of HLA-DRB1 and alanine at position 57 of HLA-DQB1 in type 1 diabetes: An epitope analysis. PLoS One. 2018;13(3):e0193684 Epub 2018/03/02. 10.1371/journal.pone.0193684 29494662 PMC5832312

[59] van Deutekom HW, Kesmir C. Zooming into the binding groove of HLA molecules: which positions and which substitutions change peptide binding most? Immunogenetics. 2015;67(8):425–36. Epub 2015/06/05. 10.1007/s00251-015-0849-y 26040913 PMC4498290

