# Supplementary figures and images for: The complex pattern of genetic associations of leprosy with HLA class I and class II alleles can be reduced to four amino acid positions
Source: PLoS Pathog. 2020 Aug 10;16(8):e1008818. doi: 10.1371/journal.ppat.1008818 (PMC7440659; doi:10.1371/journal.ppat.1008818)

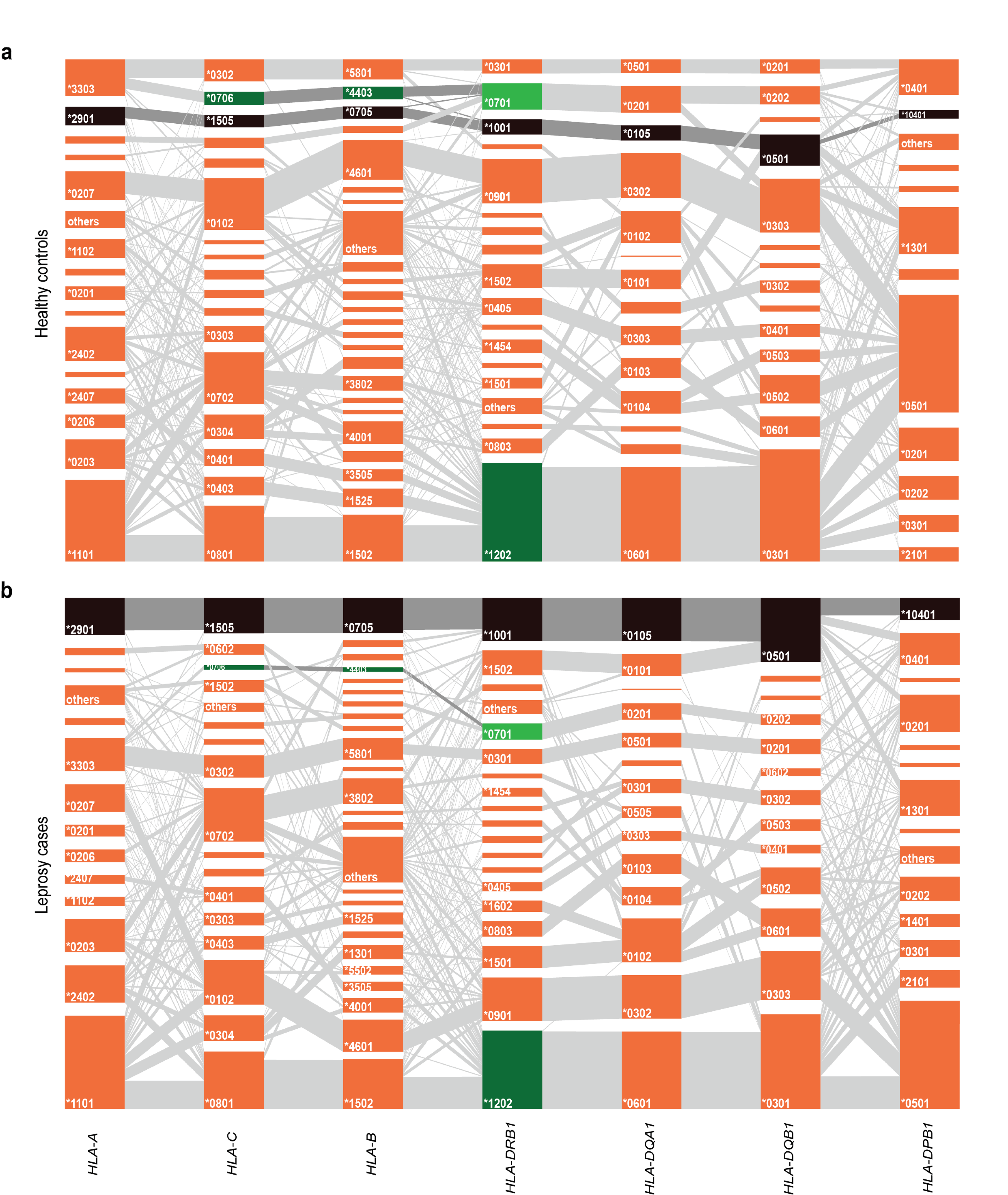

Supplement: S1 Fig — The two panels show the HLA haplotype structure in A) healthy controls (N = 468) and B) leprosy cases (N = 687). The columns represent observed alleles of seven HLA class I and class II genes, where each box corresponds to a specific four-digit HLA allele. The height of the box is relative to the observed HLA allele frequency. Alleles associated with risk or protection from leprosy per se are shown in black and green boxes, respectively (see Fig 1). Each grey line connects two HLA alleles in consecutives HLA genes, where the thickness of the line is based on the frequency of the haplotype. Haplotypes between associated alleles in consecutives genes are highlighted in darker grey. (TIF) [file ppat.1008818.s001.tif]

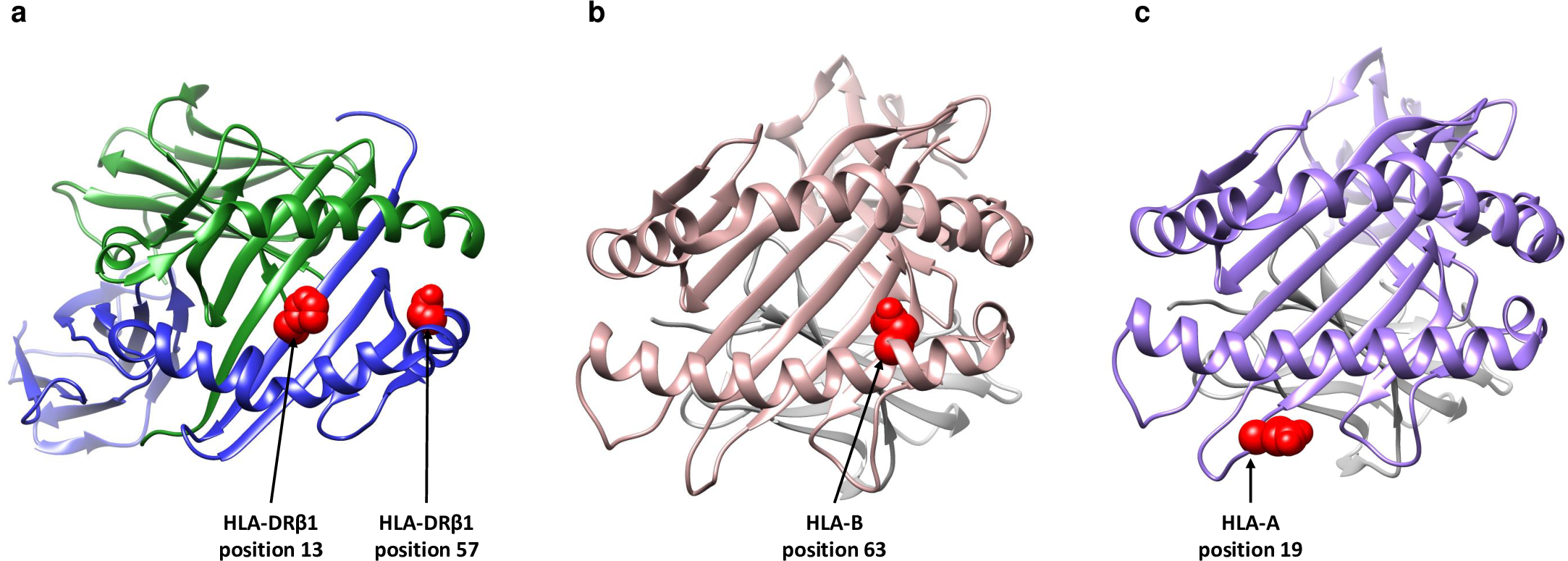

Supplement: S2 Fig — Three-dimensional ribbon representation of A) HLA-DR, B) HLA-B and C) HLA-A peptide binding grooves. HLA-DRα, HLA-DRβ1, HLA-B, HLA-A and microglobulin are shown in green, blue, pink, purple and grey, respectively. HLA-DRβ1 positions 13 and 57 (A), HLA-B position 63 (B) and HLA-A position 19 (C) are shown as spheres and highlighted in red. (TIF) [file ppat.1008818.s002.tif]
